# Efficacy and safety of intra-articular injection of mesenchymal stem cells in the treatment of knee osteoarthritis: A systematic review and meta-analysis

**DOI:** 10.1097/MD.0000000000023343

**Published:** 2020-12-04

**Authors:** Wei Ma, Cuimiao Liu, Shilu Wang, Honghao Xu, Haichao Sun, Xiao Fan

**Affiliations:** a College of Traditional Chinese Medicine, Shandong University of Traditional Chinese Medicine; b Department of Orthopedics, Affiliated Hospital of Shandong University of Traditional Chinese Medicine, Jinan; c Qingdao Municipal Hospital, 266011 Qingdao, Shandong Province; d Qingdao Huangdao District Changjiang Road Street Community Health Service Center, China.

**Keywords:** intra-articular injection, knee osteoarthritis, mesenchymal stem cells, meta-analysis

## Abstract

**Objective:**

To evaluate the effects and safety of intra-articular injection of mesenchymal stem cells on patients with knee osteoarthritis by a systematic review and meta-analysis.

**Methods:**

PubMed, EMBASE, and Cochrane Library were retrieved. An assessment of the risk of bias was done through the Cochrane Collaborative Bias Risk Tool, publication bias was assessed by plotting funnel plots and Egger tests. Pain and functional improvements in patients with knee osteoarthritis were determined by changes in VAS scores and WOMAC scores at baseline and follow-up endpoints. For the evaluation of MRI, the WORMS score and changes in cartilage volume were used. In addition, the number of adverse events in the intervention group and the control group were counted to explore the safety.

**Results:**

A total of 10 randomized controlled trials involving 335 patients were included. In the pooled analysis, compared with the control groups, the VAS scores of MSC groups decreased significantly (MD,−19.24; 95% CI: −26.31 to −12.18, *P* < .00001. All of the WOMAC scores also improved significantly: the total scores (SMD, − 0.66; 95% CI: − 1.09 to −0.23, *P* = .003), pain scores (SMD, − 0.46; 95% CI: − 0.75 to −0.17, *P* = .002), stiffness scores (SMD, −0.32; 95% CI: −0.64 to 0.00 *P* = 0.05), and functional scores (SMD, −0.36; 95% CI: −0.69 to −0.04, *P* = .03). Two studies with non-double-blind designs were the main source of heterogeneity. In terms of cartilage repair, there was no significant difference in the WORMS score, but there was a significant increase in cartilage volume in the MSC group (SMD, 0.69; 95% CI: 0.25 to 1.13, *P* = .002). The proportion of patients with adverse events in the MSCs treatment group was significantly higher than that in the control group (OR, 3.20; 95% CI: 1.50 to 6.83, *P* = .003).

**Conclusions:**

Intra-articular injection of mesenchymal stem cells is effective and safety to relieve pain and improve motor function of patients with knee osteoarthritis in a short term which is different to conclusions of previous study.

## 1 Introduction

As a degenerative disease of the synovial joints, osteoarthritis (OA) is characterized by progressive joint destruction, with clinical manifestations of joint pain and dysfunction.^[1]^ It is one of the most disabling diseases in the world,^[2–4]^ with a global prevalence of 3.8%^[5]^ that is increasing.^[6,7]^ The incidence of the subtype of knee osteoarthritis is as high as 45%, which is much higher than that of other subtypes.^[8]^ Knee OA not only leads to a reduction in the quality of life of individuals but also has an impact on the entire social health and care system, which is even considered by many as a public health crisis due to the impact of sick leave, unemployment, early retirement and treatment costs.^[3,9,10]^

Patients with knee OA may choose surgery, medication, or non-medication to relieve symptoms.^[3,11]^ Non-steroidal anti-inflammatory drugs (NSAIDs) are the main treatment in the clinic and were recommended in the clinical practice guidelines for knee OA treatment published by the American Academy of Orthopaedic Surgeons for all patients except those receiving surgical treatment.^[12]^ However, in the long run, regardless of the toxicity of the drugs themselves, long-term use of these drugs will bring serious adverse reactions to patients, such as gastrointestinal ulcers, digestive system bleeding, and cardiovascular and cerebrovascular side effects^.^^[13,14]^ Intra-articular injections of hyaluronic acid (HA), platelet-rich plasma (PRP), or corticosteroids (CC) are also some of the clinical options, but there is still much controversy regarding their efficacy or the presence of side effects.^[8,15–22]^ While surgical intervention is often advised for patients in the late stage of knee OA, although osteotomy or knee replacement can improve pain and restore knee function to some extent, the risks and complications of surgery (persistent pain and loss of function) and the possibility of further revision surgery cannot be ignored and often lead to worse clinical outcomes.^[22–26]^ From this point of view, the above treatments are only to control symptoms, not to change or delay the progression of the disease.^[8,22,27,28]^

In recent years, stem cell therapy, especially mesenchymal stem cells (MSCs), has changed the current treatment modality for KOA by providing a technique for regenerating/repairing cartilage.^[4]^ Their self-renewal ability, multi-differentiation potential, limited immunogenicity and easy cultivation and acquisition make mesenchymal stem cells popular in clinical applications.^[29–31]^ In addition, the paracrine effect of MSCs can secrete a variety of cytokines and growth factors, regulate the inclination of the intra-articular microenvironment to anti-inflammatory properties, and prevent chondrocytes from differentiating into fibrocytes and stimulate chondrocytes to produce type II collagen, thus providing help for tissue repair and regeneration.^[20,31,32]^ The clinical efficacy and safety of an intra-articular injection of mesenchymal stem cells in the treatment of knee osteoarthritis have been extensively studied in a number of clinical trials.^[26,32–35]^ Clinical outcomes include pain relief, functional recovery, and even increased cartilage volume and improvement of cartilage quality. Although some studies have explored and analyzed the effectiveness of treatment, some reports showed have shown that the clinical evidence for mesenchymal stem cells in the treatment of knee osteoarthritis is insufficient, and the outcomes of previous studies are also divergent.

Although Kim et al had studied this issue, they seemed to only get the conclusion that MSCs can alleviate pain in KOA patients, but not improve joint function and cartilage condition. In addition, they did not assess the safety of MSCs therapy. The renewal of high-quality studies on MSCs in the treatment of KOA has stimulated our interest in updating the current evidence.^[20,21,31,36,37]^ So we conducted this study to summarize all current high-quality evidence on the clinical efficacy and safety of MSCs in the treatment of KOA, and to provide a quantitative assessment. This will be very important and necessary, and the results of the study will provide evidence and guidance for the promotion and application of mesenchymal stem cell therapy in clinical practice.

## 2 Methods

### 2.1 Standards

We have designed and implemented the study in full compliance with the preferred reporting items of the system review and meta-analysis PRISMA,^[38]^ and has been registered in the PROS-

PERO database (CRD42017083426). Meanwhile, the study has been approved by the Medical Ethics Committee of Qingdao Municipal Hospital.

### 2.2 Search strategy and details

PubMed, EMBASE, and Cochrane Library were retrieved. The search was conducted on February 03, 2020, and all previous publications were retrieved, and no filters, limits, or publication date or language restrictions were enforced. The main MeSH terms used were: “Mesenchymal Stem Cells”, “Stem Cells”, “knee”, “osteoarthritis”. Further supplementary search was carried out by MeSH terms with free words. (See Table S1, Supplemental Content, which illustrates the search strategy and details). In addition, we searched retrieved studies, systematic reviews, and meta-analyses references that were cited to avoid excluding studies that met the inclusion criteria.

### 2.3 Inclusion and exclusion criteria

The inclusion criteria were as follows:

(1) Randomized controlled trial on patients with KOA;
(2) Diagnosis of KOA was based on the clinical and radiological criteria defined by the American College of Rheumatology (ACR) and illustrated degree of osteoarthritis (Kellgren-Lawrence grade);
(3) Definition of MSCs in the intervention group must meet the minimum standards set out in the International Society for Cytotherapy Consensus Statement and be administered by intra-articular injection;^[46]^
(4) One or more of the following outcome measures should be included: The Western Ontario and McMaster Universities Osteoarthritis Index (WOMAC) total score and pain, stiffness, functional subscale score; visual analog scale (VAS); the Whole-Organ Magnetic Resonance Imaging Score (WORMS); changes in cartilage volume; adverse events;

Exclusion criteria were as follows:

(1) KOA with other bone and joint diseases (Pain associated with other joints);
(2) Adjuvant surgery (arthroscopic debridement/microfracture or high/low tibial osteotomy) was used concurrently in the treatment group;
(3) Bone marrow aspirate concentrate and adipose tissue stromal vascular fraction;
(4) Research that cannot extract or transform data
(5) Unable to get full text;

### 2.4 Data extraction

Data extraction was performed independently by two authors according to a standardized form. As with the inclusion of literature into the study, disagreements that arise in the process were solved by discussion between the two authors or by consultation with a third author. The contents of the data extraction form were as follows: the first author name, year of publication, country, sample size, basic patient information (age, male-to-female ratio, body mass index), grading of osteoarthritis (Kellgren-Lawrence grade), donor source (autogenous/allogeneic), processing, culture and harvesting of cells, number of cells, immunophenotype, situation of intervention and control, follow-up, and outcome. Outcomes included clinical efficacy and safety.

### 2.5 Assessment for risk of bias and Quality of Evidence

The two authors independently assessed the overall quality and risk of bias of each included study by using the Cochrane Collaboration risk-of-bias tool.^[39]^ The contents include random sequence generation (selection bias), allocation concealment (selection bias), blinding of participants and personnel (performance bias), blinding of outcome assessment (detection bias), incomplete outcome data (attrition bias), selective reporting (reporting bias), and other biases. According to these items, each included study was scored as shaving a low, unclear, or high risk of bias. As more than 10 studies were included in this meta-analysis, it is necessary to examine the possibility of publication bias by plotting funnel plots and Egger tests. The quality of evidence was determined by 2 independent authors using the GRADEpro software (version 3.6 for Windows, GRADE Working Group). Evidence of quality is defined at four levels: high, moderate, low and very low.

### 2.6 Statistical analysis

Data analyses were performed with Review Manager software (version 5.3; Cochrane Collaboration, Oxford, UK) and Stata/mp (version 15.0; Stata Corporation, USA). If continuous outcomes were measured by the same methods and scales, they were represented and calculated using weighted mean differences (WMD) with 95% confidence intervals (CI). If not, then the standardized mean differences (SMDs) with 95% confidence intervals (CI) were selected. Heterogeneity between trials was measured by χ^2^-based Q-test and the I^2^statistic. An I^2^>50%, indicates a high degree of heterogeneity, and a random-effect model was used to merge the outcomes, and a sensitivity analysis was also performed to explore sources of heterogeneity. For an I^2^<50%, meaning that heterogeneity was not so obvious, a fixed-effect model was chosen at this time. If some data were not suitable for combined analysis, the results were described and summarized in a narrative manner. Funnel plot symmetry and the Egger test were used to verify whether there was publication bias.

## 3 Results

### 3.1 Study Characteristics

**Figure 1:**
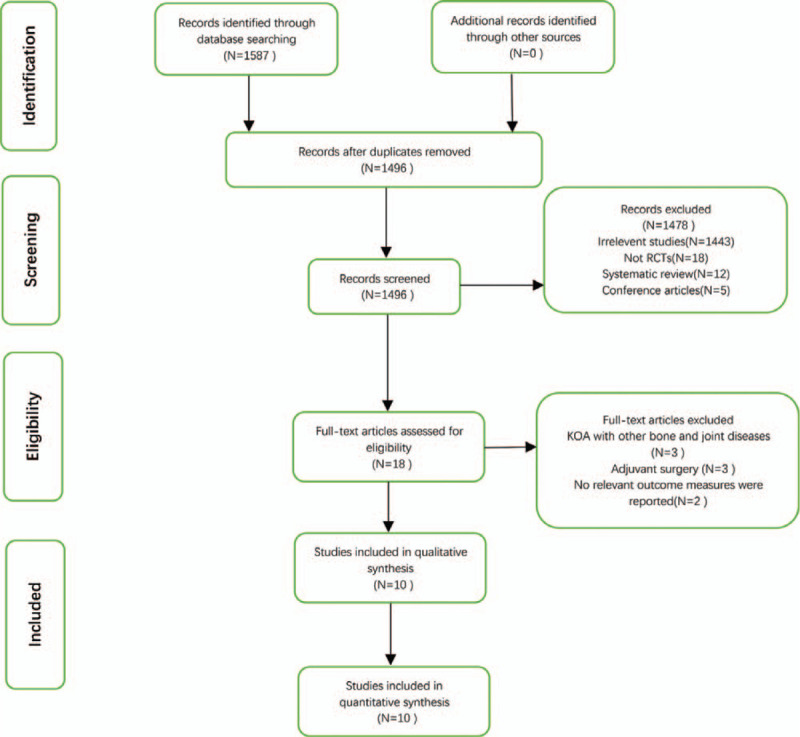
The process of literature screening in strict accordance with the inclusion/exclusion criteria. RCT, randomized controlled trial.

We found 1587 relevant articles through the literature search. After eliminating duplicates and screening titles and abstracts, 1569 articles were excluded. We conducted a full-text review of the remaining 18 articles, 8 of which were excluded (Fig. 1). A total of 10 randomized controlled trials (335 participants) were included in the meta-analysis, specific information and details were shown in Table 1. Publication intervals for all 10 articles were from 2015 to 2019. Five studies^[20,31,33,34,36]^ used autologous mesenchymal stem cells, and the remaining five studies^[26,32,35,37,40]^ used allogeneic mesenchymal stem cells. Four studies^[33–35,40]^ used bone marrow-derived mesenchymal stem cells, four studies^[20,31,32,36]^ used adipose-derived mesenchymal stem cells, and the remaining two studies used placental-derived^[26]^ and umbilical cord-derived^[37]^ mesenchymal stem cells, respectively. Hyaluronic acid (HA) was used in the control group of five studies,^[20,34,35,37,40]^ placebo in the control group of four studies,^[26,31–33]^ and conservative management in the last.^[36]^ In addition, seven of the 10 studies were followed up for 12 months, while the remaining three studies^[26,32,33]^ were for 6 months.

**Table 1:** Features of the included studies.

Study (year)	Sample Size (M:F)	Age	Bmi	Kl Grade	Blind	Intervention	Control	Donor	Immunophenotype	Dose (-106)	Outcome Measure	Follow up
Lu Liang jing (2019)	52 (6:46)	18–70	24	1–3	Double	AD -MPCs 26	HA, 26	Autologous	Positive: CD90,CD73,CD29, CD49 Negavite: CD14,CD34,CD45, HLA-DR	50 × 2	WOMAC,VAS, SF-36, others	12
LEE Woo-Suk (2019)	24 (6:18)	62.2 ± 6.5^∗^ 63.2 ± 4.2^†^	25.3 ± 4.9^∗^-<LBREAK"/>-->25.4 ± 3.0^†^	2–4	Double	AD -MSCs 12	Placbo,12	Autologous	Positive: CD90,CD73 Negavite: CD31,CD34,CD45	100	WOMAC,VAS, Knee ROM, others	6
Freitag Julien (2019)	30 (16:14)	54.6 ± 6.3^∗^-<LBREAK"/>-->51.5 ± 6.1^†^	31.6 ± 5.9^∗^-<LBREAK"/>-->25.2 ± 3.4^†^	2-3	Single (MRI)	AD -MSCs 20	Conservative Management, 10	Autologous	Positive: CD90,CD73,CD105 Negavite: CD14,CD19, CD34,CD45	100, 100 × 2	NPRS, WOMAC, KOOS, MOAKS	12
MATASJose (2019)	26 (10:16)	40-65	<30	2-3	Triple	Umbilical cord -MSCs 18	HA, 8	Allogeneic	Positive: CD90,CD73, CD105 Negavite: CD14,CD34,CD45,HLA-DR	20, 20 × 2	WOMAC,VAS, SF-36, WORMS	12
Khalifehi (2019)	20 (2:18)	35-75	<35	2–4	Double	Placenta -MSCs, 10	Placebo,10	Allogeneic	Positive: CD90,CD73, CD105 Negavite: CD31,CD34,CD45	50-60	VAS, KOOS, ROM	6
Emadedin (2018)	43 (27:16)	18–65	30	2–4	Triple	BM -MSCs, 19	Placebo,24	Autologous	Positive: CD90,CD73, CD105 Negavite: CD31,CD34, CD45	40	WOMAC,VAS, others	6
Gupta (2016)	60 (15:45)	40-70	<35	2-3	Double	BM -MSCs +HA, 40	HA,20	Allogeneic	Positive: CD90,CD73, CD44,CD166 Negavite: CD34, CD45,HLA-DR	25, 50, 75, 100	WOMAC,VAS, WORMS, others	12
Kuah (2018)	20 (12:8)	40-65	20-30	1–3	Double	AD -MSCs 16	Placebo,4	Allogeneic	Not mentioned	3.9, 6.7	WOMAC,VAS, MOAKS	12
Vega (2015)	30 (11:19)	18–75	<30	2–4	Triple	BM -MSCs, 15	HA,15	Allogeneic	Positive: CD90,CD73, CD105,CD166,CD106 Negavite: CD34,CD14,CD19,CD24 CD45,HLA-DR	40,	WOMAC,VAS, otheers	12
Lamo- Espinosa (2016)	30 (19:11)	50–80	24–32	2-3	Single (MRI)	BM -MSCs +HA, 20	HA,10	Autologous	Positive: CD90,CD73, CD44 Negavite: CD34,CD45	10, 100	WOMAC,VAS	12

AD = adipose, BM = bone marrow, BMI = body mass index, HA = hyaluronic acid, KL = Kellgren-Lawrence, MPC = mesenchymal progenitor cell, MSC = mesenchymal stem cell.

∗ Data of MSCs group.

† Data of control group.

### 3.2 Assessment for risk of bias and quality of evidence

**Figure 2:**
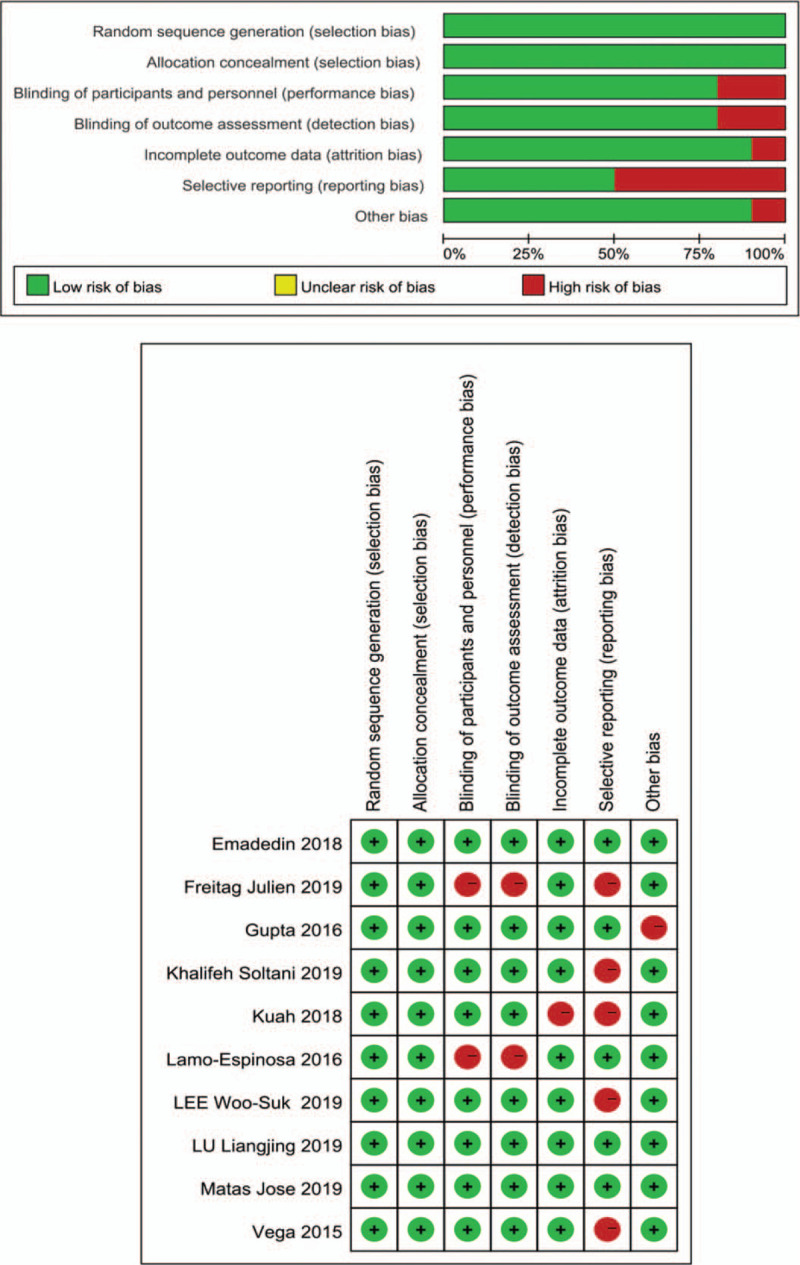
Summary of the risk of bias assessment for the included studies.

The results of the risk of bias assessment for 10 studies are shown in Figure 2. In addition, we focused on the randomization and allocation of each study. (See SupplementaryTable 2, Supplemental Content, which illustrates the summary of included studies on randomization and allocation). A total of 6 studies^[20,31,33,34,36]^ used autologous MSCs, which required mesenchymal stem cells from the patient's own adipose tissue or bone marrow in addition to the same injection process. Lamo-Espinosa et al^[34]^ and Freitag et al^[36]^ performed bone marrow or subcutaneous tissue extraction only in the intervention group, and although they both mentioned in the discussion that moral restraint prevented the same measures from being applied to the control group, both studies were defined as high risk in detection bias and performance bias. Khalifeh Soltani et al^[26]^ and Lee Woo-Suk et al^[31]^ did not fully report the data of the outcomes, although the relevant images were drawn, but we could not extract the original data and could not conduct the merged statistics, so the two studies were defined as having a high risk of reporting bias. Freitag et al,^[36]^ Vega et al^[35]^ and Kuah et al^[32]^ also reported incomplete data on total WOMAC scores and subscales (pain, stiffness, and function), and may have lacked one or more of these factors. Therefore, these three studies were defined as high risk of attrition bias. In addition, Gupta^[40]^ et al's trial became unblinded after 6 months, which was defined as high risk in other bias.

**Figure 3:**
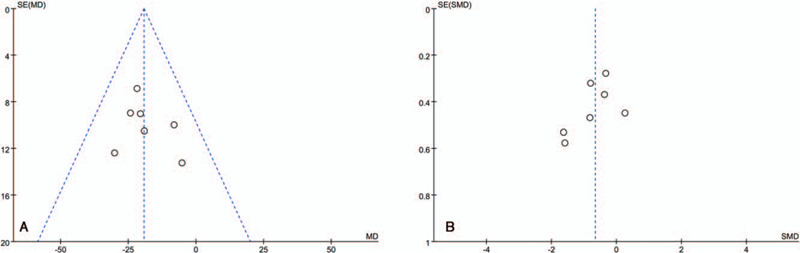
Evaluation of publication bias by funnel plot symmetry (A) Funnel plot of VAS scores (Egger, *P* = .49), (B) Funnel plot of WOMAC total scores (Egger, *P* = .22).

We plotted funnel plots for the VAS score and WOMAC total score and found no significant publication bias by examining their symmetry (Fig. 3). In addition, the Egger test by Stata software showed that the P values were 0.49 and 0.22, which means that there was no publication bias. The evidence quality of the meta-analysis was assessed by GRADEpro. Eight outcomes were evaluated separately—one high quality, two medium quality, and five low quality. (See Table S3, Supplemental Content, which illustrates the Quality grading of each outcome).

### 3.3 Visual analog scale (VAS)

**Figure 4:**
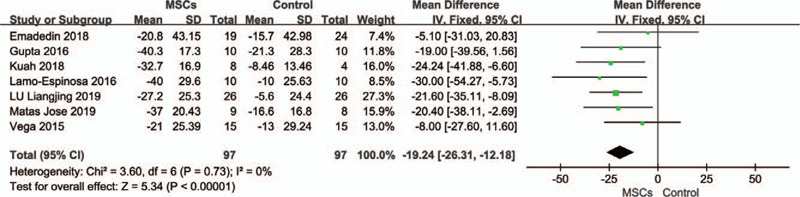
Forest plots of mean difference with 95% CI in visual analog scale (VAS) scores. Fixed-effects models were used.

A total of 7 studies^[20,32–35,37,40]^ reported VAS pain scores at baseline and final follow-up in the intervention and control groups, including 194 patients, with 97 in each group. Two studies^[26,31]^ were excluded because accurate data could not be extracted for the combined analysis, although Woo-Suk et al^[31]^ charted the data. Since the VAS by Gupta et al^[40]^ and Liangjing et al^[20]^ were different from those of other studies, to facilitate comparison, we converted them to the same scale of other studies. One study^[33]^ was followed up for 6 months and the others^[20,32,34,35,37,40]^ for 12 months. Compared with the control groups, the VAS scores of the MSCs groups decreased significantly (MD, −19.24; 95% CI: − 26.31 to − 12.18, *P* < .00001), and I^2^ = 0%, indicating that no heterogeneity exists (Fig. 4).

### 3.4 WOMAC total scores

**Figure 5:**
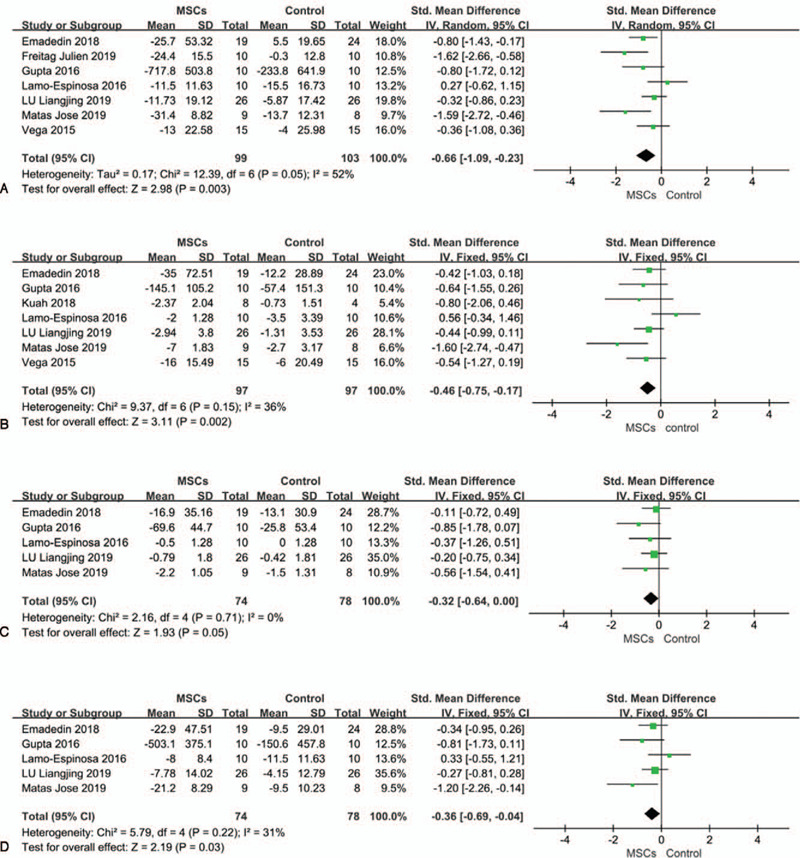
Forest plots of standardized mean difference with 95% CI in WOMAC scores. (A) WOMAC total scores. (B) WOMAC pain scores. (C) WOMAC stiffness scores. (D) WOMAC functional scores. Random effects models were used in A, Fixed-effect models were used in B, C, and D.

A total of 7 studies^[20,33–37,40]^ reported WOMAC total scores at baseline and final follow-up in the intervention and control groups, including 202 patients, 99 in the MSC groups and 103 in the control groups. One study^[33]^ was followed up for 6 months and the others^[20,34–37,40]^ for 12 months. Compared with the control group, the VAS scores of the MSC groups decreased significantly (SMD, − 0.66; 95% CI: − 1.09 to −0.23, *P* = .003), and I^2^ = 52%, indicating a high degree of heterogeneity (Fig. 5A). We tried to transform Gutpa et al's scale^[40]^ for WOMAC into the same scale as other studies, but the heterogeneity did not decrease and rose (from 52% to 61%, see Supplementary Figure 1, Supplemental Content). We used the article-by-article culling method to explore the sources of heterogeneity. When the studies by Lamo-Espinose et al^[34]^and Freitag et al^[36]^ were excluded, heterogeneity dropped to 19%. We have reason to believe that it was these two studies that led to the existence of heterogeneity, and after analyzing them and other studies, we found that in addition to these two studies, the other five studies were all double-blind or triple-blind trials.

### 3.5 WOMAC pain scores

A total of 7 studies^[20,32–35,37,40]^ reported WOMAC pain scores at baseline and final follow-up in the intervention and control groups, including194 patients, with 97 in each group. One study^[33]^ was followed up for 6 months and the others^[20,32,34,35,37,40]^ for 12 months. Compared with the control group, the WOMAC pain scores of MSC groups decreased significantly (SMD, − 0.46; 95% CI: − 0.75 to −0.17, *P* = .002), and I^2^ = 36%, indicating that a medium degree of heterogeneity exists (Fig. 5B). When the study by Lamo-Espinose et al^[34]^ was excluded, heterogeneity dropped to 0%.

### 3.6 WOMAC stiffness scores

A total of 5 studies^[20,33,34,37,40]^ reported WOMAC stiffness scores at baseline and final follow-up in the intervention and control groups, including 194 patients, with 74 in the MSC groups and 78 in the control groups. One study^[33]^ was followed up for 6 months and the others^[20,34,37,40]^ for 12 months. Compared with the control group, the WOMAC stiffness scores of the MSC groups decreased significantly (SMD, −0.32; 95% CI: −0.64 to 0.00, *P* = .05), and I^2^ = 0% (Fig. 5C).

### 3.7 WOMAC functional scores

A total of 5 studies^[20,33,34,37,40]^ reported WOMAC functional scores at baseline and final follow-up in the intervention and control groups, including194 patients, with 74 in the MSC groups and 78 in the control groups. One study^[33]^ was followed up for 6 months and the others^[20,34,37,40]^ for 12 months. Compared with the control group, the WOMAC functional scores of the MSC groups decreased significantly (SMD, −0.36; 95% CI: −0.69 to −0.04, *P* = .03), and I^2^ = 31%, indicating that a medium degree of heterogeneity exists. After excluding the study by Lamo-Espinose et al,^[34]^ heterogeneity decreased to 2% (Fig. 5D).

### 3.8 MRI evaluation

Since the included studies had high heterogeneity in the evaluation of MRI, we decided to merge groups according to different evaluation methods and then conduct statistical analysis.

#### 3.8.1 WORMS Scores

**Figure 6:**
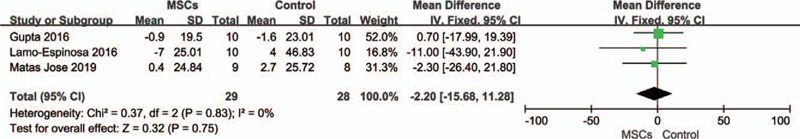
Forest plots of mean difference with 95% CI in WORMS scores. Fixed-effects models were used.

Three studies^[34,37,40]^ used WORMS scores to assess the final MRI results, including 57 patients, with 29 in the MSCs group and 28 in the control group. All studies were followed up for 12 months. Treatment with MSCs led to improved MRI outcomes (MD, −2.20; 95% CI: −15.68 to 11.28, *P* = .75), although this was not statistically significant, and I^2^ = 0% (Fig. 6).

#### 3.8.2 Cartilage Volume

**Figure 7:**
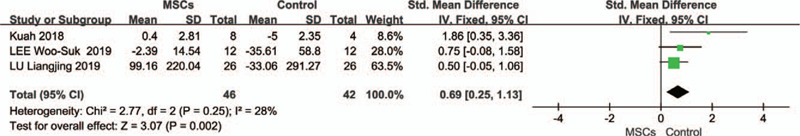
Forest plots of standardized mean difference with 95% CI in changes in cartilage volume. Fixed-effect effects models were used.

Three studies^[20,31,32]^ used the changes in cartilage volume to assess final MRI results, including 88 patients, with 46 in the MSC group and 42 in the control group. All studies were followed up for 12 months. Since the evaluation scale of LEE Woo-Suk et al^[31]^ was different from that of the other two studies, we converted it to the same scale for comparison. Compared with the control group, the changes in cartilage volume of the MSC groups showed significant improvements (SMD, 0.69; 95% CI: 0.25 to 1.13, *P* = .002), and I^2^ = 28%, indicating that a mild degree of heterogeneity exists (Fig. 7).

Khalifeh Soltani et al^[26]^ evaluated the MRI outcomes by measuring 28 measurement points of each patient's knee joint. The results showed that the cartilage thickness of the MSC group increased significantly, while the control group had no change. Freitag et al^[36]^ evaluated outcomes using MOAKS (MRI Osteoarthritis Knee Scores), and 67% of the patients in the control group had progressive cartilage loss and 56% had prolonged osteophyte formation. In the MSC group, 89% of patients could observe cartilage improvement or cartilage loss without progression, and 89% of patients showed no progress in osteophyte formation. Vega et al^[35]^ evaluated the quality of cartilage in patients with T2 relaxation measurements.

### 3.9 Adverse events (AEs)

All 10 of the studies were evaluated for adverse events during treatment and follow-up. Most of the adverse events were mild and moderate, and the clinical symptoms were joint pain, swelling, pain at the injection site, and joint effusion. Only four SAEs (grade ≥ 4) were reported. Gupta et al^[40]^ reported three severe adverse events from three different dose MSCs groups: 25 M (dyslipidemia), 50 M (anemia), 150 M (muscle hemorrhage), complete recovery after symptomatic treatment. Kuah et al.^[32]^ reported a severe adverse event in which one patient in the MSC group developed severe prepatellar bursitis 13 days after the injection, and the symptoms were alleviated after 2 weeks of treatment. The investigators considered the event to be related to the joint injection technique.

**Figure 8:**
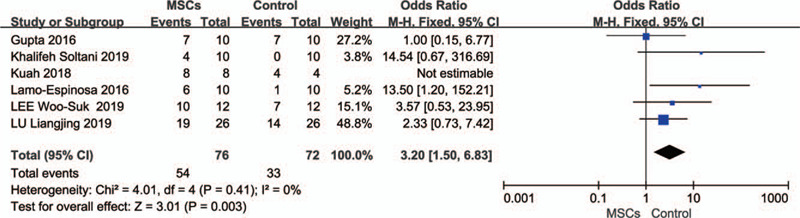
Forest plots of odds ratios with 95% CI in number of patients with adverse events. Fixed-effects models were used.

Since each patient was likely to experience multiple adverse events, we conducted a combined analysis of only studies that clearly stated the number of patients who experienced adverse events during treatment and follow-up. A total of 6 studies^[20,26,31,32,34,40]^ reported the number of patients with adverse events in the intervention and control groups, including 148 patients, 76 in the MSC groups and 72 in the control groups. Two studies^[26,31]^ were followed up for 6 months and the others^[20,32,34,40]^ for 12 months. The proportion of patients with adverse events in the MSC treatment group was significantly higher than that in the control group (OR, 3.20; 95% CI: 1.50 to 6.83, *P* = .003), and I^2^ = 0% (Fig. 8).

## 4 Discussion

As a new treatment mode, cell therapy has changed the paradigm that traditional treatment cannot reverse or delay the progress of knee osteoarthritis and has attracted extensive attention in the medical community.^[41]^ First, as a pluripotent stem cell, it has all the commonalities of stem cells, namely, the self-renewal and multidirectional differentiation abilities. Their constant self-renewal and ability to differentiate into osteoblasts and chondrocytes under specific conditions illustrate their great potential in tissue repair and regeneration.^[42,43]^ Second, MSCs acquire paracrine and immunomodulatory effects by releasing cytokines and growth factors, thereby manipulating the microenvironment of the knee joint and stimulating local growth and reducing the immune response.^[44,45]^ This may be very beneficial for knee osteoarthritis, which is characterized by a degenerative and inflammatory pathophysiology.^[11]^ The clinical efficacy and safety of MSCs have been extensively studied, but most of them are of low quality and have insufficient evidence or are controversial. In this study, we systematically analyzed 10 randomized controlled trials to evaluate the clinical efficacy and safety of an intra-articular injection of mesenchymal stem cells. The strength of this study lies first in its comprehensiveness, which is a summary of all high-quality studies today. Secondly, it has strict inclusion and exclusion criteria. Studies with concomitant treatment were excluded, such as low/high tibial osteotomy, microfracture, knee replacement. In addition, we evaluated the cell adherence, cell immunophenotype and cell differentiation ability of the included studies according to the MSC criteria defined by the Mesenchymal Stem Cell Committee of the International Society for Cell Therapy (ISCT) to improve the uniformity and effectiveness of this study.^[46]^

Our meta-analysis yielded several new findings. At short-term follow-up (6–12 months), patients treated with MSCs had significantly lower VAS scores (*P* < .00001) and had a new, smaller range of confidence intervals than previous study by Kim et al,^[27]^ suggesting a new and more reliable outcome. Second, the total WOMAC score and various subscale scores (pain, stiffness, and function) of patients treated with MSCs were also significantly lower than those of patients in the control group (all *P* values were less than .05). Unlike Kim et al^[27]^ who previously reported only significant improvements in pain, simultaneous significant improvements in pain and function were a completely new finding. We believe that it is the addition of newly incorporated studies of adipose tissue and umbilical cord sources that have led us to different results. This is one of the reasons why we insist on doing this research. In the evaluation of MRI, due to the heterogeneity of evaluation methods, we cannot pool and analyze the data on a large scale. Fortunately, we still got some encouraging results. A significant increase in cartilage volume was observed in the MSC group compared with the control group (*P* = .002). In terms of worms score, although the difference between the MSC group and control group is not significant (*P* = .75), it is undeniable that the MSC group does show a trend of score improvement. Several other studies^[26,35,36]^ have also shown a trend toward improved imaging in the MSCs treatment group. Although these results are insufficient to draw a conclusion that MSCs can repair defects, by comparison with the control group, we at least have reason to believe that they can delay the degeneration of articular cartilage or even terminate it.

With the in-depth study of MSCs therapy, the choice of the best donor source and the optimal dose has become an important issue. Mesenchymal stem cells have a wide range of sources, and the donor sources commonly used in clinical research are bone marrow, adipose tissue, placenta and umbilical cord. Initially, people tended to choose bone marrow mesenchymal stem cells for culture and expansion. Later, it was found that compared with bone marrow, adipose tissue was more easily accessible, the isolation process was simple, the yield was higher, and it had the same chondrogenic potential.^[4,47]^ In recent years, studies on umbilical cord-derived MSCs have shown that they have stronger proliferation and migration ability than bone-marrow-derived MSCs^[26,37,48]^ and secrete more chondrogenic factors. The clinical selection of less invasive, more easily harvested adipose tissue and umbilical cord mesenchymal stem cells may be a better choice. Similarly, the choice of the source of autologous or allogeneic cells is also a matter worthy of discussion. Theoretically, compared with autologous MSCs, allogeneic MSCs may have adverse reactions such as tumorigenesis and host immune rejection.^[49]^ However, no serious adverse events have been reported as to whether this is the case in the included studies or previous studies.^[50]^ This may be related to the immune privilege of MSCs.^[51,52]^ The allogeneic mesenchymal stem cells should be more promising in the absence of significant differences in clinical efficacy and incidence of adverse events, and this makes it possible to produce off-the-shelf products.^[53]^ It cannot only reduce the pain suffered by patients during treatment but can also save time and reduce treatment costs. The cost of stem cell therapy is certainly higher compared to the conventional therapies that have been widely promoted, such as non-steroidal anti-inflammatory drugs and hyaluronic acid. This is not only reflected in its long way to go in universality. It is also reflected in the technology itself, cell extraction, expansion, transport, and so on. Meanwhile, it costs much less than traditional surgery, such as osteotomy or knee replacement and so on. Therefore, after the technology has matured, timing of application of cell therapy and the balance of clinical efficacy and cost is still a big challenge for clinicians. But we believe that the emergence of more research, including our study, will reduce its cost to acceptable standards for most people.

The choice of the best clinical dose is usually determined by clinical efficacy and safety. Some previous studies seem to indicate that the incidence of adverse events in the high-dose group is slightly higher than that in the low-dose group,^[8,40]^ and the reason for this may be that the rapid circulation of synovial fluid or injection of large doses of cells into the knee joint leads to apoptosis, resulting in joint pain, swelling, and other symptoms.^[54]^ However, we cannot jump to conclusions until higher quality or more convincing evidence emerges. More important is that this problem enlightens us that efficacy should be balanced with safety when promoting the clinical universality of MSC therapy because patients with KOA are mostly elderly people who usually take a variety of drugs or suffer from various underlying diseases. Therefore, weighing the actual clinical problems in formulating reasonable dosage is still a major test for clinicians.

This study has several limitations. First, caution should be exercised in interpreting the results. Although we have tried our best to avoid the impact of concomitant surgical treatment on efficacy and have tightly controlled the criteria for MSCs, heterogeneity among different studies still exists. It is mainly manifested in the differences in cell preparation (extraction, expansion, culture, and harvest) and transportation. Therefore, the standardization of the process is an urgent problem to be solved in the future. Second, all of the studies that we included were administered by intra-articular injection. Some studies have found that MSC implantation through open or arthroscopic surgery may be more conducive to cartilage repair,^[55]^ while scaffold-based MSC transplantation may better regenerate the anterior cruciate ligament and meniscus.^[56]^ Although we cannot compare different modes of administration, the great advantage that intra-articular injection can be administered directly in the outpatient setting is sufficient to make it a more universal option. Shortening the treatment time and reducing the cost of treatment can also save patients from additional suffering. Third, we included five studies that included patients with Kellgren-Lawrence grade 4 knee osteoarthritis. At the most advanced stage of the disease, we are not sure whether the course of the disease can be delayed or even reversed, especially with autologous-derived MSCs. Although, Lamo et al^[34]^ showed in their study that they were able to obtain sufficient cell numbers in all patients regardless of age and knee OA grade. However, with the aging of the human body, the self-renewal and differentiation ability of MSCs is significantly reduced, specifically, the potential of MSCs in patients with OA is lower than that in healthy people.^[57]^ Whether or not there is an impact on clinical efficacy or the magnitude of the impact is an unexplained question by current evidence. Nevertheless, we have aggregated the latest evidence and obtained new outcomes that are different from those of previous studies. These results demonstrate the effectiveness of an intraarticular injection of MSCs in the treatment of osteoarthritis of the knee.

## 5 Conclusion

Our study shows that an intra-articular injection of mesenchymal stem cells can relieve pain and improve function in patients with knee osteoarthritis in a short term and is relatively safe. Although current evidence is insufficient to conclude that MSCs can repair cartilage defects, we at least have reason to believe that they have a protective effect on cartilage and delay articular cartilage degradation. These results suggest that MSC therapy has great potential in the future, but more homogeneous RCT studies are needed to validate it.

## Acknowledgments

We thank AJE (https://www.aje.cn) for English language editing.

## Author contributions

**Conceptualization:** Wei Ma, Shilu Wang, Honghao Xu, Haichao Sun, Xiao Fan.

**Data curation:** Wei Ma, Cuimiao Liu, Shilu Wang, Honghao Xu, Xiao Fan.

**Formal analysis:** Wei Ma, Cuimiao Liu, Shilu Wang, Honghao Xu, Haichao Sun.

**Funding acquisition:** Xiao Fan.

**Investigation:** Wei Ma, Shilu Wang, Honghao Xu, Haichao Sun.

**Methodology:** Wei Ma, Cuimiao Liu, Honghao Xu, Haichao Sun, Xiao Fan.

**Project administration:** Xiao Fan.

**Resources:** Wei Ma, Shilu Wang, Xiao Fan.

**Software:** Wei Ma, Cuimiao Liu, Shilu Wang, Honghao Xu.

**Supervision:** Honghao Xu, Xiao Fan.

**Validation:** Wei Ma, Cuimiao Liu, Xiao Fan.

**Visualization:** Wei Ma, Cuimiao Liu, Xiao Fan.

**Writing – original draft:** Wei Ma, Shilu Wang.

**Writing – review & editing:** Wei Ma, Cuimiao Liu, Honghao Xu, Xiao Fan.

## Supplementary Material

Supplemental Digital Content

## Supplementary Material

Supplemental Digital Content

## Supplementary Material

Supplemental Digital Content

## Supplementary Material

Supplemental Digital Content
