# Supplementary material for: Efficacy and safety of intra-articular injection of mesenchymal stem cells in the treatment of knee osteoarthritis: A systematic review and meta-analysis
Source: Medicine (Baltimore). 2020 Dec 4;99(49):e23343. doi: 10.1097/MD.0000000000023343 (PMC7717742; doi:10.1097/MD.0000000000023343)
Supplement: Supplemental Digital Content [file medi-99-e23343-s001.docx]

Supplemental Content

TableS1. Search strategy and details.

| **Table S1 Source: PUBMED (searched on: February 03, 2020)** | | | | |
| --- | --- | --- | --- | --- |
| **Search** | | **Query** | **Items found** | |
| #14 | | Search #7 AND #10 AND #13 | 519 | |
| #13 | | Search #11 OR #12 | 92897 | |
| #12 | | Search (((((((((((Osteoarthritides[Title/Abstract]) OR Osteoarthrosis[Title/Abstract]) OR Osteoarthroses[Title/Abstract]) OR Arthritis, Degenerative[Title/Abstract]) OR Arthritides, Degenerative[Title/Abstract]) OR Degenerative Arthritides[Title/Abstract]) OR Degenerative Arthritis[Title/Abstract]) OR Arthrosis[Title/Abstract]) OR Arthroses[Title/Abstract]) OR Osteoarthrosis Deformans[Title/Abstract])) OR osteoarthritis[Title/Abstract] | 74804 | |
| #11 | | Search "Osteoarthritis"[Mesh] | 61622 | |
| #10 | | Search #8 OR #9 | 141874 | |
| #9 | | Search knee[Title/Abstract] | 138202 | |
| #8 | | Search "Knee"[Mesh] | 14018 | |
| #7 | | Search #3 OR #6 | 350797 | |
| #6 | | Search ("Stem Cells"[Mesh]) OR stem cell*[Title/Abstract] | 341314 | |
| #5 | | Search stem cell*[Title/Abstract] | 258246 | |
| #4 | | Search "Stem Cells"[Mesh] | 208625 | |
| #3 | | Search #1 OR #2 | 70757 | |
| #2 | | Search ((((((((((((((((((((((((((((((((((Stem Cell, Mesenchymal[Title/Abstract]) OR Stem Cells, Mesenchymal[Title/Abstract]) OR Mesenchymal Stem Cell[Title/Abstract]) OR Bone Marrow?Mesenchymal Stem Cells[Title/Abstract]) OR Bone Marrow Stromal Cells[Title/Abstract]) OR Bone Marrow Stromal Cell[Title/Abstract]) OR Bone Marrow Stromal Cells, Multipotent[Title/Abstract]) OR Multipotent Bone Marrow Stromal Cells[Title/Abstract]) OR Adipose-Derived Mesenchymal Stem Cells[Title/Abstract]) OR Adipose Derived Mesenchymal Stem Cells[Title/Abstract]) OR Mesenchymal Stem Cells, Adipose-Derived[Title/Abstract]) OR Mesenchymal Stem Cells, Adipose Derived[Title/Abstract]) OR Adipose-Derived Mesenchymal Stromal Cells[Title/Abstract]) OR Adipose Derived Mesenchymal Stromal Cells[Title/Abstract]) OR Adipose Tissue-Derived Mesenchymal Stem Cells[Title/Abstract]) OR Adipose Tissue Derived Mesenchymal Stem Cells[Title/Abstract]) OR Adipose Tissue-Derived Mesenchymal Stromal Cells[Title/Abstract]) OR Adipose Tissue Derived Mesenchymal Stromal Cells[Title/Abstract]) OR Mesenchymal Stromal Cells[Title/Abstract]) OR Mesenchymal Stromal Cell[Title/Abstract]) OR Stromal Cell, Mesenchymal[Title/Abstract]) OR Stromal Cells, Mesenchymal[Title/Abstract]) OR Multipotent Mesenchymal Stromal Cells[Title/Abstract]) OR Mesenchymal Stromal Cells, Multipotent[Title/Abstract]) OR Mesenchymal Progenitor Cell[Title/Abstract]) OR Mesenchymal Progenitor Cells[Title/Abstract]) OR Progenitor Cell, Mesenchymal[Title/Abstract]) OR Progenitor Cells, Mesenchymal[Title/Abstract]) OR Wharton Jelly Cells[Title/Abstract]) OR Wharton's Jelly Cells[Title/Abstract]) OR Wharton's Jelly Cell[Title/Abstract]) OR Whartons Jelly Cells[Title/Abstract]) OR Bone Marrow Stromal Stem Cells[Title/Abstract])) OR mesenchymal stem cell*[Title/Abstract] | 67830 | |
| #1 | | Search "Mesenchymal Stem Cells"[Mesh] | 35310 | |
|  | |  |  | |
| **Source: EMBASE (searched on: February 03, 2020)** | | | | |
| **Search** | **Query** | | | **Items found** |
| #56 | Search #39 AND #42 AND #55 | | | 905 |
| #55 | Search #43 OR #44 OR #45 OR #46 OR #47 OR #48 OR #49 OR #50 OR #51 OR #52 OR #53 OR #54 | | | 149109 |
| #54 | Search ‘osteoarthrosis deformans':ab,ti | | | 155 |
| #53 | Search ‘arthroses':ab,ti | | | 600 |
| #52 | Search ‘arthrosis':ab,ti | | | 7298 |
| #51 | Search ’degenerative arthritis':ab,ti | | | 1522 |
| #50 | Search ’degenerative arthritides':ab,ti | | | 14 |
| #49 | Search ’arthritides, degenerative':ab,ti | | | 0 |
| #48 | Search ’arthritis, degenerative':ab,ti | | | 69 |
| #47 | Search ’osteoarthroses':ab,ti | | | 36 |
| #46 | Search ’osteoarthrosis':ab,ti | | | 4139 |
| #45 | Search ’osteoarthritides':ab,ti | | | 3 |
| #44 | Search ’osteoarthritis':ab,ti | | | 88282 |
| #43 | Search ’osteoarthritis'/exp | | | 128605 |
| #42 | Search #40 OR #41 | | | 192661 |
| #41 | Search ’knee':ab,ti | | | 177716 |
| #40 | Search ’knee'/exp | | | 67893 |
| #39 | Search #35 OR #38 | | | 474998 |
| #38 | Search #36 OR #37 | | | 462512 |
| #37 | Search ’stem cell':ab,ti | | | 231641 |
| #36 | Search **'**stem cell'/exp | | | 362650 |
| #35 | #1 OR #2 OR #3 OR #4 OR #5 OR #6 OR #7 OR #8 OR #9 OR #10 OR #11 OR #12 OR #13 OR #14 OR #15 OR #16 OR #17 OR #18 OR #19 OR #20 OR #21 OR #22 OR #23 OR #24 OR #25 OR #26 OR #27 OR #28 OR #29 OR #30 OR #31 OR #32 OR #33 OR #34 | | | 77646 |
| #34 | Search ’bone marrow stromal stem cells':ab,ti | | | 289 |
| #33 | Search ’whartons jelly cells':ab,ti | | | 1 |
| #32 | Search ’wharton s jelly cell':ab,ti | | | 1 |
| #31 | Search ’wharton s jelly cells':ab,ti | | | 36 |
| #30 | Search ’wharton jelly cells':ab,ti | | | 4 |
| #29 | Search ’progenitor cells, mesenchymal':ab,ti | | | 54 |
| #28 | Search ’progenitor cell, mesenchymal':ab,ti | | | 5 |
| #27 | Search ’mesenchymal progenitor cells':ab,ti | | | 1177 |
| #26 | Search ’mesenchymal progenitor cell':ab,ti | | | 230 |
| #25 | Search ’mesenchymal stromal cells, multipotent':ab,ti | | | 2 |
| #24 | Search ’multipotent mesenchymal stromal cells':ab,ti | | | 874 |
| #23 | Search ’stromal cells, mesenchymal':ab,ti | | | 47 |
| #22 | Search ’stromal cell, mesenchymal':ab,ti | | | 5 |
| #21 | Search ’mesenchymal stromal cell':ab,ti | | | 1560 |
| #20 | Search ’mesenchymal stromal cells':ab,ti | | | 8802 |
| #19 | Search ’adipose tissue derived mesenchymal stromal cells':ab,ti | | | 118 |
| #18 | Search ’adipose tissue-derived mesenchymal stromal cells':ab,ti | | | 118 |
| #17 | Search ’adipose tissue derived mesenchymal stem cells':ab,ti | | | 1012 |
| #16 | Search ’adipose tissue-derived mesenchymal stem cells':ab,ti | | | 1012 |
| #15 | Search ’adipose derived mesenchymal stromal cells':ab,ti | | | 172 |
| #14 | Search ’adipose-derived mesenchymal stromal cells':ab,ti | | | 172 |
| #13 | Search ’mesenchymal stem cells, adipose derived':ab,ti | | | 28 |
| #12 | Search ’mesenchymal stem cells, adipose-derived':ab,ti | | | 28 |
| #11 | Search ’adipose derived mesenchymal stem cells':ab,ti | | | 1834 |
| #10 | Search ’adipose-derived mesenchymal stem cells':ab,ti | | | 1834 |
| #9 | Search ’multipotent bone marrow stromal cells':ab,ti | | | 16 |
| #8 | Search ’bone marrow stromal cells, multipotent':ab,ti | | | 1 |
| #7 | Search ’bone marrow stromal cell':ab,ti | | | 1608 |
| #6 | Search ’bone marrow stromal cells':ab,ti | | | 7297 |
| #5 | Search ’bone marrow mesenchymal stem cells':ab,ti | | | 7480 |
| #4 | Search ’mesenchymal stem cell':ab,ti | | | 11992 |
| #3 | Search ’stem cells, mesenchymal':ab,ti | | | 268 |
| #2 | Search ’stem cell, mesenchymal':ab,ti | | | 29 |
| #1 | Search ’mesenchymal stem cell'/exp | | | 56704 |

| **Source: Cochrane Library (searched on: February 03, 2020)** | | |
| --- | --- | --- |
| **Search** | **Query** | **Items found** |
| #1 | MeSH descriptor: [Osteoarthritis] explode all trees | 7167 |
| #2 | MeSH descriptor: [Knee] explode all trees | 769 |
| #3 | (osteoarthritides):ti,ab,kw | 0 |
| #4 | (Osteoarthrosis):ti,ab,kw | 477 |
| #5 | (Osteoarthroses):ti,ab,kw | 2 |
| #6 | (Arthritis, Degenerative):ti,ab,kw | 247 |
| #7 | (Arthritides, Degenerative):ti,ab,kw | 0 |
| #8 | (Degenerative Arthritides):ti,ab,kw | 0 |
| #9 | (Degenerative Arthritis):ti,ab,kw | 247 |
| #10 | (Arthrosis):ti,ab,kw | 602 |
| #11 | (Arthroses):ti,ab,kw | 56 |
| #12 | (Osteoarthrosis Deformans):ti,ab,kw | 5 |
| #13 | (Osteoarthritis):ti,ab,kw | 16772 |
| #14 | (knee):ti,ab,kw | 27988 |
| #15 | #3 OR #4 OR #5 OR #6 OR #7 OR #8 OR #9 OR #10 OR #11 OR #12 OR #13 | 17256 |
| #16 | #15 OR #1 | 17256 |
| #17 | #14 OR #2 | 27988 |
| #18 | #16 AND #17 | 11574 |
| #19 | (mesenchymal stem cell):ti,ab,kw | 1474 |
| #20 | (stem cell):ti,ab,kw | 13182 |
| #21 | #19 OR #20 | 13182 |
| #22 | #18 AND #21 | 163 |
