# Supplementary material for: Efficacy and safety of intra-articular injection of mesenchymal stem cells in the treatment of knee osteoarthritis: A systematic review and meta-analysis
Source: Medicine (Baltimore). 2020 Dec 4;99(49):e23343. doi: 10.1097/MD.0000000000023343 (PMC7717742; doi:10.1097/MD.0000000000023343)
Supplement: Supplemental Digital Content [file medi-99-e23343-s002.docx]

Supplemental Content

| Table S2 Summary of included studies on randomization and allocation | | |
| --- | --- | --- |
| Study | Random sequence generation | Allocation concealment |
| Lu Liangjing 2019 | All the patients enrolled were arranged to take liposuction, and autologous MPCs were prepared. Central randomization was performed by a biostatistician using PROC PLAN in SAS and executed in GMP workshop. | The preparation for injection and the IA injection were performed in two different clean rooms by a trained experienced investigator, who was separate from the evaluator. A curtain was used to prevent patients from seeing the injection procedures. All study-related case report forms recorded only the randomization number. |
| LEE Woo-Suk 2019 | According to the randomization, participants were blindly assigned to AD-MSCs injection (MSC group) or normal saline injection (control group). | Neither the physician nor the patient was aware of who was receiving AD-MSCs, |
| Freitag Julien 2019 | Participant group allocation was prepared in advance using a web-based automated random number generator. | Participants were not blinded to their treatment allocation. |
| MATAS Jose 2019 | Randomized allocation by electronic data entry system. | All injections were performed by two orthopedic surgeons in the study, blinded to the component being injected,in syringes equal in volume (3 cc) and external aspect. |
| Emadedin 2018 | Block randomization method (block size of four) based on the Consolidated Standards of Reporting Trials (CONSORT) protocol. | The laboratory expert then provided the orthopedist with similar vials that were labeled with the patient name and registration code. The vials contained either MSCs or placebo to be implanted based on the assigned random code. |
| Gupta 2016 | using a computer generated randomization. | It was not possible to distinguish between Stempeucel® and placebo upon visual inspection of the blinded syringes. |
| Khalifeh Soltani 2019 | Twenty patients with symptomatic knee OA were randomly put into a group (A or B), which was defined by a statistician using a random table | The barrel of the all injection syringes had been completely covered. |
| Kuah 2018 | A statistician not directly involved in the conduct of the study prepared the randomization schedule using a block method. | A screen was used to ensure the patient remained blinded to the treatment allocation. |
| Lamo-Espinosa 2016 | Participants were assigned to comparison groups by an unblinded computer-generated list, based on unrestricted randomization, which was maintained centrally by staff with no clinical involvement in the trial. | No center knew the treatment allocation of any patient until  the patient had been recruited into the trial. |
| Vega 2015 | The recruited patients were block randomized by the quality control manager of the cell production facility, who was blinded, to receive either the control or the experimental treatment. | NA |
|  |  |  |
