# Supplementary material for: Efficacy and safety of intra-articular injection of mesenchymal stem cells in the treatment of knee osteoarthritis: A systematic review and meta-analysis
Source: Medicine (Baltimore). 2020 Dec 4;99(49):e23343. doi: 10.1097/MD.0000000000023343 (PMC7717742; doi:10.1097/MD.0000000000023343)
Supplement: Supplemental Digital Content [file medi-99-e23343-s003.docx]

Supplemental Content

| Table S3  Quality grading of each outcome | | | | |
| --- | --- | --- | --- | --- |
| Outcomes | Absolute  efect (95% CI) | Relative  efect (95% CI) | Number of  patients (studies) | Quality of the  evidence (GRADE) |
| VAS | MD 19.24 lower (26.31 to 12.18 lower) | NA | 194(7) | ⊕⊕⊕⊕ HIGH |
| WOMAC total | SMD 0.66 lower (1.09 to 0.23 lower) | NA | 202(7) | ⊕⊕⊕O MODERATE |
| WOMAC pain | SMD 0.46 lower (0.75 to 0.17 lower) | NA | 194(7) | ⊕⊕⊕O MODERATE |
| WOMAC stiffness | SMD 0.32 lower (0.64 lower to 0 higher) | NA | 152(5) | ⊕⊕OO LOW |
| WOMAC function | SMD 0.36 lower (0.69 to 0.04 lower) | NA | 152(5) | ⊕⊕OO LOW |
| WORMS | MD 2.2 lower (15.68 lower to 11.28 higher) | NA | 57(3) | ⊕⊕OO LOW |
| Cartilage volume | SMD 0.69 higher (0.25 to 1.13 higher) | NA | 88(3) | ⊕⊕OO LOW |
| Adverse events | NA | OR 3.2 (1.5 to 6.83) | 148(6) | ⊕⊕OO LOW |
