# Supplementary material for: Efficacy and safety of intra-articular injection of mesenchymal stem cells in the treatment of knee osteoarthritis: A systematic review and meta-analysis
Source: Medicine (Baltimore). 2020 Dec 4;99(49):e23343. doi: 10.1097/MD.0000000000023343 (PMC7717742; doi:10.1097/MD.0000000000023343)
Supplement: Supplemental Digital Content [file medi-99-e23343-s004.docx]

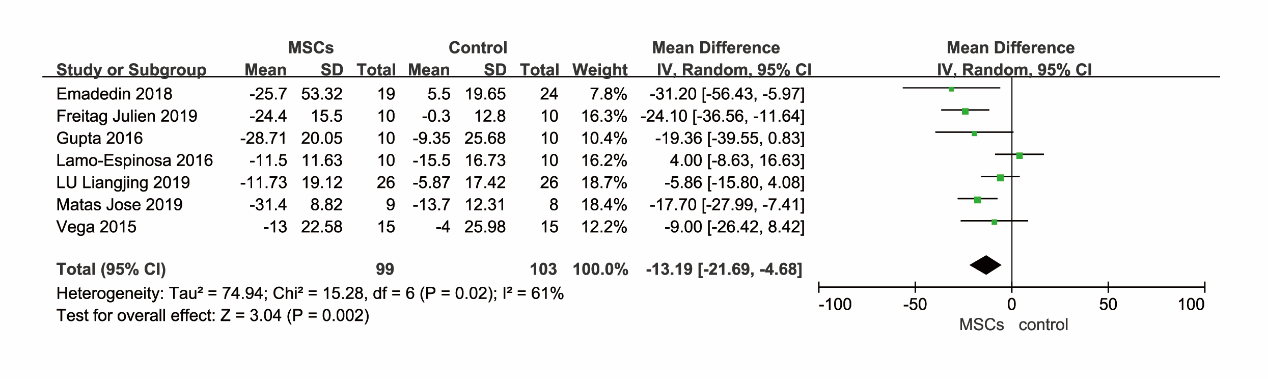


**Figure S1**. Forest plots of mean differences with 95% CI in WOMAC total scores. Fixed-effects models were used (Unified Outcome Scale).
